# Pattern of OPD utilisation during the COVID-19 pandemic under the Universal Coverage Scheme in Thailand: what can 850 million records tell us?

**DOI:** 10.1186/s12913-023-09121-3

**Published:** 2023-02-03

**Authors:** Jarawee Sukmanee, Rukmanee Butchon, Picharee Karunayawong, Thanayut Saeraneesopon, Chulathip Boonma, Yot Teerawattananon, Wanrudee Isaranuwatchai

**Affiliations:** 1 Department of Health Ministry of Public Health, Health Intervention and Technology Assessment Program (HITAP), 6Th Floor, 6Th Building, Nonthaburi, Thailand; 2 Saw Swee Hock School of Public Health, National University of Singapore, Singapore, Singapore; 3 Health Administration Division, Office of the Permanent Secretary of the Ministry of Public Health, Nonthaburi, Thailand; 4 Institute of Health Policy, Management and Evaluation, University of Toronto, Toronto, Canada

**Keywords:** COVID-19, Interrupted time-series, Lockdown, Outpatient department, Thailand, Universal coverage scheme

## Abstract

**Background:**

Out-patient department (OPD) is a crucial component of the healthcare systems in low- and middle-income countries including Thailand. A considerable impact of coronavirus disease 2019 (COVID-19) pandemic and its control measures, especially the lockdown, on utilisation of OPD services was expected. This study thus aims to estimate the pattern of OPD utilisation during the COVID-19 pandemic in Thailand including overall utilisation and within each sub-groups including diagnostic group, age group, and health region.

**Methods:**

This study was a secondary data analysis of aggregated outpatient data from patients covered under the Universal Coverage Scheme (UCS) in Thailand over a 4-year period (2017–2020). Interrupted time series analyses and segmented Quasi-Poisson regression were used to examine the impact of COVID-19 on the overall OPD utilisation including the impact on each diagnostic group, age groups, health regions, and provinces.

**Results:**

Analysis of 845,344,946 OPD visits in this study showed a seasonal pattern and increasing trend in monthly OPD visits before the COVID-19 pandemic. A 28% (rate ratio (RR) 0.718, 95% confidence interval (CI): 0.631–0.819) and 11% (RR 0.890, 95% CI: 0.811–0.977) reduction in OPD visits was observed during the lockdown and post-lockdown periods, respectively, when compared to the pre-lockdown period. Diseases of respiratory system were most affected with a RR of 0.411 (95% CI: 0.320–0.527), while the number of visits for non-communicable diseases (ICD-10: E00–E90, I00–I99) and elderly (> 60 years) dropped slightly. The post-lockdown trend in monthly OPD visits gradually increased to the pre-pandemic levels in most groups.

**Conclusions:**

Thailand’s OPD utilisation rate during the COVID-19 lockdown decreased in some diseases, but the service for certain group of patients appeared to remain available. After the COVID-19 lockdown, the rate returned to the pre-pandemic level in a timely manner. Equipped with a knowledge of OPD utilisation pattern during COVID-19 based on a national real-world database could aid with a better preparation of healthcare system for future pandemics.

**Supplementary Information:**

The online version contains supplementary material available at 10.1186/s12913-023-09121-3.

## Background

The pandemic of coronavirus disease 2019 (COVID-19) has been the latest threat to global health since the first case was reported in China in late December 2019 [1]. Thailand, an upper middle-income country located in South-East Asia and not far from China, had rather highly effective response at the start of the COVID-19 pandemic, which began in March 2020, and experienced relatively few COVID-19 cases compared to other countries during the start of pandemic [1]. This pattern was contributed mainly due to several non-pharmaceutical measures (NPI) implemented during the beginning of COVID-19 pandemic including isolation and quarantine of detected cases, use of face masks and hand hygiene, and social distancing as well as full-scale national lockdown from March 26 to May 3, 2020 (i.e., night curfews, travel ban, and closures of school and all public spaces) [2]. As out-patient department (OPD) is a crucial component for improved service delivery and efficiency enhancement specially in context of healthcare systems in low- and middle-income countries (LMICs) [3, 4], the impact of COVID-19 pandemic and its measures on utilisation of same was expected to be substantial. For instance, studies in Kenya and China showed that the number of OPD visits had dropped by approximately 35–60% during the early period of pandemic [5, 6].

Thailand has three main health care schemes including the Civil Servant Medical Benefit Scheme (CSMBS), Social Security Scheme (SSS), and Universal Coverage Scheme (UCS) [7]. The UCS, established since 2002, is being managed by the National Health Security Office (NHSO) [8] and covered approximately 80% of the population in Thailand or 48 million people in 2017. Therefore, analysing the pattern of OPD utilisation during the COVID-19 pandemic using the UCS’ national database from NHSO could aid with the planning for future and predict the readiness on pandemic preparedness response. This study aims to estimate the pattern of OPD utilisation during COVID-19 in Thailand, which included overall utilisation and utilisation in each diagnostic group, age group, and health region.

## Methods

This study was a secondary data analysis of outpatient data from patients covered under the UCS in Thailand. Data were obtained from the NHSO over a 4-year period between January 2017 and December 2020 which was separated into 3 periods: 1) the pre-pandemic period (January 2017 to March 2020); 2) the lockdown (April – May 2020); and 3) the post-lockdown period (June – December 2020). The number of patients for each month were aggregated into 22 diagnostic groups (based on the 10^th^ revision International Classification of Diseases [ICD-10]) (Additional file 1) [9]; 7 age groups (10-year age bands from 0 to 60 and older than 60 years); and 13 health regions throughout Thailand [10]. The OPD utilisation was represented by the number of OPD visits divided by number of UCS beneficiaries in each group. The study was approved by the ethics committee of Institute for the Development of Human Research Protections.

### Statistical Analysis

An interrupted time series (ITS) analysis was performed to estimate the impact of COVID-19 on pattern of OPD utilisation in Thailand. The impact was modelled using a segmented Quasi-Poisson regression with pre-pandemic trends (January 2017 – March 2020) and indicator variables for lockdown (April–June 2020) and post-lockdown periods (July – December 2020) as follows:

$${Y}_{t}={\beta }_{0}+{\beta }_{1}T+{\beta }_{2}{X}_{t}+{\beta }_{3}T{X}_{t}+\epsilon$$

where ${X}_{t}$ represented the dummy variable indicating the pre-pandemic (coded 0), lockdown (coded 1) or post-lockdown periods (coded 2), *T* was time in months from January 2017, and ${Y}_{t}$ was the OPD utilisation at time *t*. In the model, ${\beta }_{0}$ estimates the baseline level at *T* = 0, ${\beta }_{1}$ estimates the change in outcome associated with a time unit increase, ${\beta }_{2}$ estimates the level change following the intervention, and ${\beta }_{3}$ is the slope change following the interaction between time and intervention.

The rate ratios (RRs) and their 95% confidence interval (95% CI) of OPD utilisation during lockdown and post-lockdown periods compared to OPD utilisation in the pre-pandemic period were calculated. The RRs for overall OPD utilisation as well as for each diagnostic group, age groups, health regions, and provinces were explored. All statistical analyses were performed using R version 4.1.3 (R Foundation for Statistical Computing, Vienna, Austria) [11]. An ITS was conducted using The packages lmtest [12], vcd [13], Epi [14], tsModel [15], and splines. A *p*-value of 0.05 or lower was considered statistically significant (i.e. significant change).

## Results

**Fig. 1:**
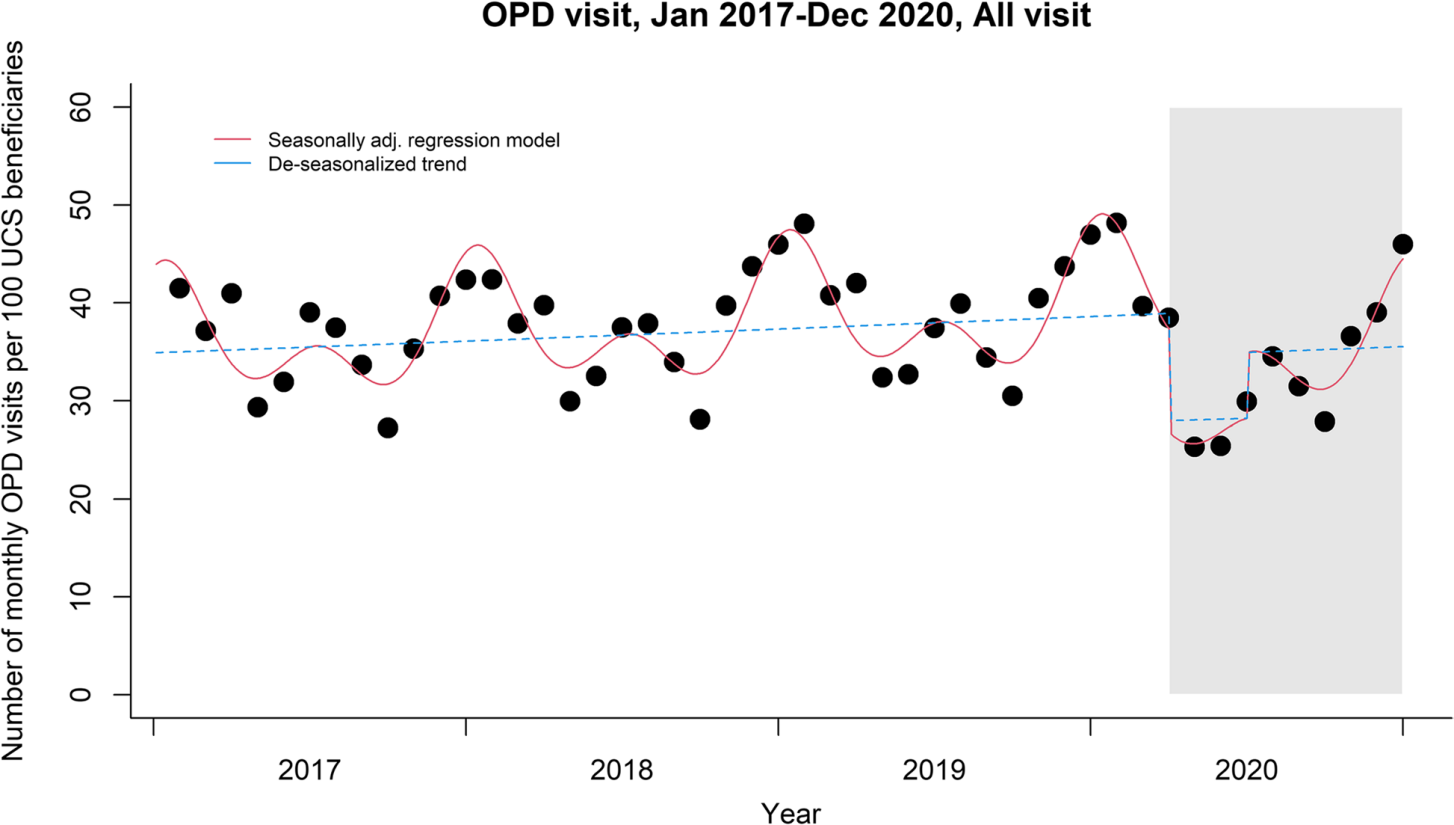
Number of monthly OPD visits per 100 UCS beneficiaries from January 2017 to December 2020. Note: Black dots = average number of monthly OPD visits per 100 UCS beneficiaries from January 2017 to December 2020; Red solid lines = seasonally adjusted trend of monthly OPD visits per 100 UCS beneficiaries based on the regression model; Blue dashed lines = de-seasonalized trend of monthly OPD visits per 100 UCS beneficiaries

A total of 845,344,946 OPD visits was observed during January 2017 to December 2020. There was an increasing trend in monthly OPD visits per 100 UCS beneficiaries across the pre-pandemic period (Fig. 1).

The seasonal patterns were also observed with the highest and lowest number of OPD visits occurring in January and September, respectively. The number of OPD visits decreased by 28% (RR 0.718, 95% CI: 0.631–0.819) and 11% (RR 0.890, 95% CI: 0.811–0.977) during the COVID-19 lockdown and post-lockdown periods, accordingly, when compared to the pre-pandemic period.

The relative change in OPD visits during and after COVID-19 lockdown in each diagnostic group is shown in Table 1 and Additional file 2.

**Table 1:** Relative change in OPD visits during and after COVID-19 lockdown, stratified by diagnostic groups

Diagnostic groups (ICD-10)	Lockdown		Post-lockdown	
	**RR**	**95% CI**	**RR**	**95% CI**
J00-J99: Diseases of the respiratory system	0.411	(0.320–0.527)	0.637	(0.551–0.737)
P00-P96: Certain conditions originating in the perinatal period	0.489	(0.322–0.743)	0.638	(0.477–0.854)
U00-U99: Codes for special purposes	0.516	(0.430–0.620)	0.802	(0.711–0.904)
K00-K93: Diseases of the digestive system	0.545	(0.476–0.624)	0.787	(0.718–0.862)
Q00-Q99: Congenital malformations, deformations and chromosomal abnormalities	0.650	(0.530–0.797)	0.883	(0.763–1.022)
M00-M99: Diseases of the musculoskeletal system and connective tissue	0.670	(0.593–0.757)	0.888	(0.814–0.968)
A00-B99: Certain infectious and parasitic diseases	0.703	(0.629–0.786)	0.788	(0.725–0.856)
Z00-Z99: Factors influencing health status and contact with health services	0.730	(0.581–0.916)	0.955	(0.815–1.120)
H00-H59: Diseases of the eye and adnexa	0.735	(0.655–0.824)	0.890	(0.817–0.969)
R00-R99: Symptoms, signs and abnormal clinical and laboratory findings, not elsewhere classified	0.749	(0.689–0.815)	0.824	(0.774–0.877)
G00-G99: Diseases of the nervous system	0.753	(0.680–0.834)	0.893	(0.828–0.962)
L00-L99: Diseases of the skin and subcutaneous tissue	0.762	(0.699–0.832)	0.892	(0.835–0.954)
S00-T98: Injury, poisoning and certain other consequences of external causes	0.790	(0.727–0.859)	0.973	(0.913–1.038)
F00-F99: Mental and behavioural disorders	0.797	(0.722–0.878)	0.837	(0.776–0.902)
H60-H95: Diseases of the ear and mastoid process	0.803	(0.727–0.887)	0.895	(0.830–0.966)
D50-D89: Diseases of the blood and blood-forming organs and certain disorders involving the immune mechanism	0.806	(0.731–0.888)	0.864	(0.802–0.931)
E00-E90: Endocrine, nutritional and metabolic diseases	0.832	(0.738–0.938)	0.935	(0.854–1.024)
I00-I99: Diseases of the circulatory system	0.843	(0.759–0.937)	0.922	(0.851–1.000)
C00-D48: Neoplasms	0.856	(0.756–0.970)	0.896	(0.814–0.985)
N00-N99: Diseases of the genitourinary system	0.868	(0.798–0.945)	0.944	(0.885–1.008)
V01-Y98: External causes of morbidity and mortality	0.922	(0.681–1.249)	0.923	(0.729–1.168)
O00-O99: Pregnancy, childbirth and the puerperium	0.932	(0.866–1.003)	0.983	(0.929–1.041)

Lockdown = April–June 2020; post-lockdown = July–December 2020; *CI* Confidence interval

There was a significant decline in the number of OPD visits for all diagnostic groups during the lockdown period, except for ICD-10 codes of V01–Y98 (external causes of morbidity and mortality such as transport accidents or assault) and O00–O99 (pregnancy, childbirth, and the puerperium). The largest drop in OPD utilisation was observed in diseases of the respiratory system (ICD-10: J00–J99) followed by certain conditions originating in the perinatal period (P090–P96) with RRs of 0.411 (95% CI: 0.320–0.527) and 0.489 (95% CI: 0.322–0.743), respectively. A significant decline in the number of OPD visits due to respiratory diseases after the lockdown remained until the end of 2020 (RR 0.637, 95% CI: 0.551–0.737). Meanwhile, the numbers of OPD visits for endocrine, nutritional, and metabolic diseases (E00–E90), diseases of the circulatory system (I00–I99), and diseases of the genitourinary system (N00–N99) during the post-lockdown period were not significantly different from the pre-pandemic period.

During the lockdown period, the number of OPD visits in all age groups was significantly decreased (Table 2 and Additional file 3).

**Table 2:** Relative change in OPD visits during and after COVID-19 lockdown, stratified by age groups

Age group	Lockdown		Post-lockdown	
	**RR**	**95% CI**	**RR**	**95% CI**
0–10 years	0.545	(0.419–0.709)	0.822	(0.691–0.978)
11–20 years	0.534	(0.454–0.627)	0.806	(0.724–0.897)
21–30 years	0.775	(0.692–0.869)	0.902	(0.829–0.983)
31–40 years	0.787	(0.681–0.911)	0.915	(0.824–1.016)
41–50 years	0.774	(0.667–0.899)	0.912	(0.819–1.015)
51–60 years	0.778	(0.679–0.891)	0.915	(0.829–1.010)
> 60 years	0.801	(0.696–0.922)	0.936	(0.844–1.038)

Lockdown = April–June 2020; post-lockdown = July–December 2020; *CI* Confidence interval

The younger the age groups, the higher the impact was observed. For example, a drop by almost half was detected in 0–10 age groups with a RR of 0.545 (95% CI: 0.419–0.709), whereas a drop of only 20% (RR 0.801, 95% CI: 0.696–0.922) was observed in a group of 60 years and above. The number of OPD visits after the lockdown in the age group of older than 30 years was similar to their pre-pandemic levels while the age group of 30 years or younger reported significantly lower than their pre-pandemic levels.

Table 3 and Additional file 4 demonstrate the relative change in OPD visits during and after COVID-19 lockdown in 13 health regions across the country [10]. Thailand has 77 provinces which were grouped into 13 health regions. Each health region has between four to eight provinces with one exception for health region 13 which includes only 1 province, Bangkok, the capital (and biggest province) of Thailand.

**Table 3:** Relative change in OPD visits during and after COVID-19 lockdown, stratified by health regions

Health region	Lockdown		Post-lockdown	
	**RR**	**95% CI**	**RR**	**95% CI**
1	0.720	(0.614–0.844)	0.921	(0.823–1.031)
2	0.786	(0.695–0.889)	0.928	(0.850–1.013)
3	0.749	(0.663–0.847)	0.903	(0.827–0.986)
4	0.757	(0.672–0.852)	0.861	(0.789–0.940)
5	0.684	(0.608–0.770)	0.889	(0.819–0.966)
6	0.718	(0.621–0.830)	0.864	(0.778–0.959)
7	0.773	(0.678–0.881)	0.909	(0.826–0.999)
8	0.744	(0.644–0.860)	0.895	(0.807–0.991)
9	0.705	(0.575–0.865)	0.924	(0.798–1.070)
10	0.725	(0.612–0.857)	0.937	(0.831–1.056)
11	0.589	(0.484–0.716)	0.796	(0.697–0.909)
12	0.686	(0.590–0.797)	0.844	(0.759–0.937)
13	0.901	(0.498–1.629)	0.759	(0.452–1.273)

Lockdown = April–June 2020; post-lockdown = July–December 2020; *CI* Confidence interval

The number of monthly OPD visits in health region 13 (Bangkok, the capital of Thailand) was approximately 10 visits per 100 UCS beneficiaries when compared to 40 visits in other health regions. A significant decrease in the number of in OPD visits during lockdown period was observed in all health regions, except in health region 13 (Bangkok). The strongest impact of the lockdown was noted in health region 11 (southern provinces), specifically the number of OPD visits fell by 10–20% after the lockdown when compared to the pre-pandemic level. There was no significant difference in the number of OPD visits after the lockdown in health regions 1 and 2 (northern provinces), 9 and 10 (northeastern provinces), and 13 (Bangkok).

## Discussion

This analysis of a big data on OPD visits in Thailand showed an impact of the COVID-19 lockdown on ambulatory service utilisation. The minimal impact can be seen for planned visits as the number of visits among non-communicable diseases and in elderly were only trivially dropped. On the contrary, the higher impact was observed for diseases of respiratory system, which can be corelated to a decreased incidence due to social distancing measures. The findings reassured that the services for those patients who have been planned in advanced were only slightly affected during the lockdown period. After the lockdown, the number of OPD visits returned to their pre-pandemic levels in most diagnostic groups, age groups, and health regions.

The minimal impact of the COVID-19 lockdown on OPD visits among adults and elderly was observed in our study with a decline rate of approximately 20–25% whereas a declining rate in children and adolescent (0–10 and 10–20 years) was around 45%. Moreover, the observed impact of COVID-19 lockdown appeared to be similar across health regions with one exception of health region 13 which is Bangkok, the capital of Thailand. This variation might be due to the differences between the capital and other parts of the country such as the highest population density for the capital and the health service structure of Bangkok [16]. The varying degree of declines for each service was also reported in other countries including Ethiopia, Haiti, Ghana, Lao People’s Democratic Republic, Mexico, Nepal, South Africa, Chile, and South Korea [17]. Moreover, the number of OPD visits in most diagnostic groups, age groups, and health regions returned to the pre-pandemic levels once the lockdown was lifted. A slight impact on the planned OPD visits might be due to a relatively small outbreak of COVID-19 throughout 2020. For instance, a total of 4,862 COVID-19 cases and 62 deaths were reported in 2020 [18]. The estimated excess all-cause deaths associated with COVID-19 were -22 (95% CI: -34 to -11) in 2020 compared with 44 (95% CI: 26–62) in 2021–2022 [19].

The COVID-19 control measures had a positive effect on the incidence of respiratory diseases especially during the lockdown period. This trend was also observed in Singapore and the United States [20, 21]. However, the difference in magnitude might be due to variation in stringency index in each country [22]. In our study, the effect of COVID-19 control measures on respiratory diseases was persisted long after the lockdown, conversely, a re-emergence of respiratory viral infections was found in Singapore after 13 weeks of the lockdown [21]. A high proportion of population wearing mask and washing their hands in Thailand despite lockdown easing [23] and a higher population density in Singapore [24] might explain the results.

To date, there are a few studies evaluating the impact of COVID-19 on OPD utilisation nationally [5, 6, 17, 25]. We analysed more than 800 million OPD records over 4-year period in this study, which covered ~ 80% of the population in Thailand. Knowing the OPD utilisation pattern during the COVID-19 pandemic could be used to answer health policy-related questions. For example, a positive effect of COVID-19 control measures on respiratory diseases could support a continuation of mask wearing in public spaces.

There are limitations to our study since we used an aggregated data. First, demographic and clinical characteristics information, except age group and health region, were not available for confounding adjustment. Second, diagnoses in each ICD-10 diagnostic group could not be specified. We also could not explore adverse health outcomes (i.e., hospitalisation rate, mortality rate) after a reduction in OPD utilisation due to lack of relevant data. Finally, the current study only focused on the first year of the pandemic; therefore, future research should explore and compare the OPD utilisation patterns in subsequent years including detailed types of visits (e.g., emergency cases) to support further planning for our healthcare system.

In conclusion, we found that the OPD utilisation rate in Thailand decreased during the COVID-19 lockdown with a varying impact on different service and returned to pre-pandemic levels after the lockdown. The results from this study suggest that healthcare providers might have considered both policy and pandemic situation when implementing control measures for each health sector. Further studies on healthcare providers’ attitude and behaviour toward the lockdown policy could provide a better understanding of which service sector should be prioritised and strengthen the healthcare system for future pandemics.

## Supplementary Information

**Additional file 1:** **Supplementary Table 1.** International Statistical Classification of Diseases and Related Health Problems 10th Revision (ICD-10) chapters.

**Additional file 2:** Number of monthly OPD visits per 100 UCS beneficiaries from January 2017 to December 2020, stratified by diagnostic groups.

**Additional file 3:** Number of monthly OPD visits per 100 UCS beneficiaries from January 2017 toDecember 2020, stratified by age groups.

**Additional file 4:** Number of monthly OPD visits per 100 UCS beneficiaries from January 2017 to December 2020, stratified by health regions.

## Data Availability

The datasets generated and/or analysed during the current study are not publicly available based on the confidentiality agreement to access the data with the data custodian, but general information on the database is available from the corresponding author on reasonable request.

## Abbreviations

ICD: International Classification of Diseases
ITS: Interrupted time series
NHSO: National Health Security Office
OPD: Out-patient department
UCS: Universal Coverage Scheme

## References

[1] World Health Organization. Archived: WHO Timeline - COVID-19. 2020. https://www.who.int/news/item/27-04-2020-who-timeline---covid-19. Accessed 24 Mar 2022.

[2] Rajatanavin N, Tuangratananon T, Suphanchaimat R, Tangcharoensathien V (2021). Responding to the COVID-19 second wave in Thailand by diversifying and adapting lessons from the first wave. BMJ Glob Health.

[3] Starfield B (2012). Primary care: an increasingly important contributor to effectiveness, equity, and efficiency of health services. SESPAS report 2012. Gac Sanit.

[4] Rohde J, Cousens S, Chopra M, Tangcharoensathien V, Black R, Bhutta ZA (2008). 30 years after Alma-Ata: has primary health care worked in countries?. The Lancet.

[5] Xiao H, Dai X, Wagenaar BH, Liu F, Augusto O, Guo Y (2021). The impact of the COVI D-19 pandemic on health services utilization in China: Time-series analyses for 2016–2020. Lancet Reg Health - West Pac..

[6] Wambua S, Malla L, Mbevi G, Kandiah J, Nwosu A-P, Tuti T (2022). Quantifying the indirect impact of COVID-19 pandemic on utilisation of outpatient and immunisation services in Kenya: a longitudinal study using interrupted time series analysis. BMJ Open.

[7] World Bank. The World Bank In Thailand. 2022. https://www.worldbank.org/en/country/thailand. Accessed 24 Jul 2022.

[8] Tangcharoensathien V, Witthayapipopsakul W, Panichkriangkrai W, Patcharanarumol W, Mills A (2018). Health systems development in Thailand: a solid platform for successful implementation of universal health coverage. Lancet Lond Engl.

[9] World Health Organization. ICD-10 Version:2010. 2010. https://icd.who.int/browse10/2010/en. Accessed 25 Jul 2022.

[10] National Health Security Office. NHSO Regional Office. 2020. https://eng.nhso.go.th/view/1/NHSO_Regional_Office/EN-US. Accessed 24 Jul 2022.

[11] R Core Team (2021). A language and environment for statistical computing..

[12] Zeileis A, Hothorn T (2002). Diagnostic checking in regression relationships. R News.

[13] Meyer D, Zeileis A, Hornik K (2021). vcd: Visualizing categorical data.

[14] Carstensen B, Plummer M, Laara E, Hills M (2022). Epi: A package for statistical analysis in epidemiology.

[15] Peng RD (2022). McDermott with contributions from A. tsModel: Time series modeling for air pollution and health.

[16] Health Systems Research Institute, National Health Security Office Region 13 Bangkok. Recommendations on the development of health service management in Bangkok: Special health region. 2018. https://kb.hsri.or.th/dspace/handle/11228/5035.

[17] Arsenault C, Gage A, Kim MK, Kapoor NR, Akweongo P, Amponsah F (2022). COVID-19 and resilience of healthcare systems in ten countries. Nat Med.

[18] Department of Disease Control, Ministry of Public Health. DDC OPENDATA COVID-19 Thailand. 2022. https://covid19.ddc.moph.go.th/en. Accessed 18 Apr 2022.

[19] World Health Organization. Global excess deaths associated with COVID-19 (modelled estimates). 2021. https://www.who.int/data/sets/global-excess-deaths-associated-with-covid-19-modelled-estimates. Accessed 24 Aug 2022.

[20] Partridge E, McCleery E, Cheema R, Nakra N, Lakshminrusimha S, Tancredi DJ (2021). Evaluation of seasonal respiratory virus activity before and after the statewide COVID-19 shelter-in-place order in Northern California. JAMA Netw Open.

[21] Wan WY, Thoon KC, Loo LH, Chan KS, Oon LLE, Ramasamy A (2021). Trends in respiratory virus infections during the COVID-19 pandemic in Singapore, 2020. JAMA Netw Open.

[22] Hale T, Angrist N, Goldszmidt R, Kira B, Petherick A, Phillips T (2021). A global panel database of pandemic policies (Oxford COVID-19 Government Response Tracker). Nat Hum Behav.

[23] Oxford Policy Management, United Nations. Social impact assessment of COVID-19 in Thailand. 2020. https://www.unicef.org/thailand/reports/social-impact-assessment-covid-19-thailand. Accessed 20 Jul 2022.

[24] World Bank. Population density (people per sq. km of land area). 2022. https://data.worldbank.org/indicator/EN.POP.DNST?view=chart. Accessed 5 Sep 2022.

[25] Barasa E, Kazungu J, Orangi S, Kabia E, Ogero M, Kasera K (2021). Indirect health effects of the COVID-19 pandemic in Kenya: a mixed methods assessment. BMC Health Serv Res.

