# Supplementary material for: Pattern of OPD utilisation during the COVID-19 pandemic under the Universal Coverage Scheme in Thailand: what can 850 million records tell us?
Source: BMC Health Serv Res. 2023 Feb 3;23:116. doi: 10.1186/s12913-023-09121-3 (PMC9897880; doi:10.1186/s12913-023-09121-3)
Supplement: Supplementary file 1 — Additional file 1: Supplementary Table 1. International Statistical Classification of Diseases and Related Health Problems 10th Revision (ICD-10) chapters. [file 12913_2023_9121_MOESM1_ESM.docx]

**Supplementary Table 1** International Statistical Classification of Diseases and Related Health Problems 10th Revision (ICD-10) chapters

| **Chapter** | **Block** | **Title** |
| --- | --- | --- |
| I | A00–B99 | Certain infectious and parasitic diseases |
| II | C00–D48 | Neoplasms |
| III | D50–D89 | Diseases of the blood and blood-forming organs and certain disorders involving the immune mechanism |
| IV | E00–E90 | Endocrine, nutritional and metabolic diseases |
| V | F00–F99 | Mental and behavioural disorders |
| VI | G00–G99 | Diseases of the nervous system |
| VII | H00–H59 | Diseases of the eye and adnexa |
| VIII | H60–H95 | Diseases of the ear and mastoid process |
| IX | I00–I99 | Diseases of the circulatory system |
| X | J00–J99 | Diseases of the respiratory system |
| XI | K00–K93 | Diseases of the digestive system |
| XII | L00–L99 | Diseases of the skin and subcutaneous tissue |
| XIII | M00–M99 | Diseases of the musculoskeletal system and connective tissue |
| XIV | N00–N99 | Diseases of the genitourinary system |
| XV | O00–O99 | Pregnancy, childbirth and the puerperium |
| XVI | P00–P96 | Certain conditions originating in the perinatal period |
| XVII | Q00–Q99 | Congenital malformations, deformations and chromosomal abnormalities |
| XVIII | R00–R99 | Symptoms, signs and abnormal clinical and laboratory findings, not elsewhere classified |
| XIX | S00–T98 | Injury, poisoning and certain other consequences of external causes |
| XX | V01–Y98 | External causes of morbidity and mortality |
| XXI | Z00–Z99 | Factors influencing health status and contact with health services |
| XXII | U00–U99 | Codes for special purposes |
