# Supplementary material for: Pattern of OPD utilisation during the COVID-19 pandemic under the Universal Coverage Scheme in Thailand: what can 850 million records tell us?
Source: BMC Health Serv Res. 2023 Feb 3;23:116. doi: 10.1186/s12913-023-09121-3 (PMC9897880; doi:10.1186/s12913-023-09121-3)
Supplement: Supplementary file 2 — Additional file 2: Number of monthly OPD visits per 100 UCS beneficiaries from January 2017 to December 2020, stratified by diagnostic groups. [file 12913_2023_9121_MOESM2_ESM.pptx]

## Slide 1
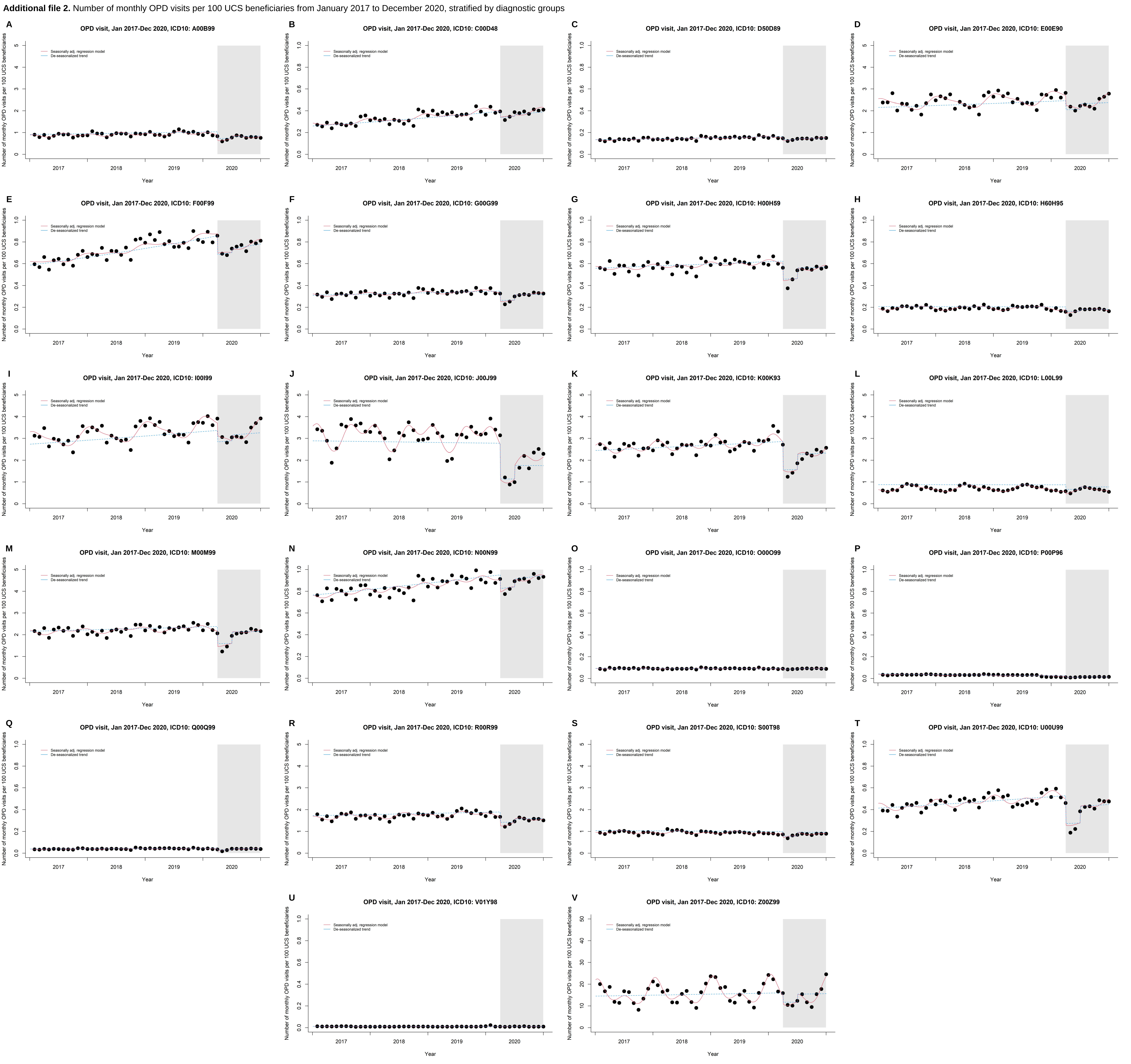

Additional file 2. Number of monthly OPD visits per 100 UCS beneficiaries from January 2017 to December 2020, stratified by diagnostic groups
B
C
D
A
E
F
G
H
I
J
K
L
M
N
O
P
Q
R
S
T
U
V
