# Supplementary material for: Pattern of OPD utilisation during the COVID-19 pandemic under the Universal Coverage Scheme in Thailand: what can 850 million records tell us?
Source: BMC Health Serv Res. 2023 Feb 3;23:116. doi: 10.1186/s12913-023-09121-3 (PMC9897880; doi:10.1186/s12913-023-09121-3)
Supplement: Supplementary file 3 — Additional file 3: Number of monthly OPD visits per 100 UCS beneficiaries from January 2017 toDecember 2020, stratified by age groups. [file 12913_2023_9121_MOESM3_ESM.pptx]

## Slide 1
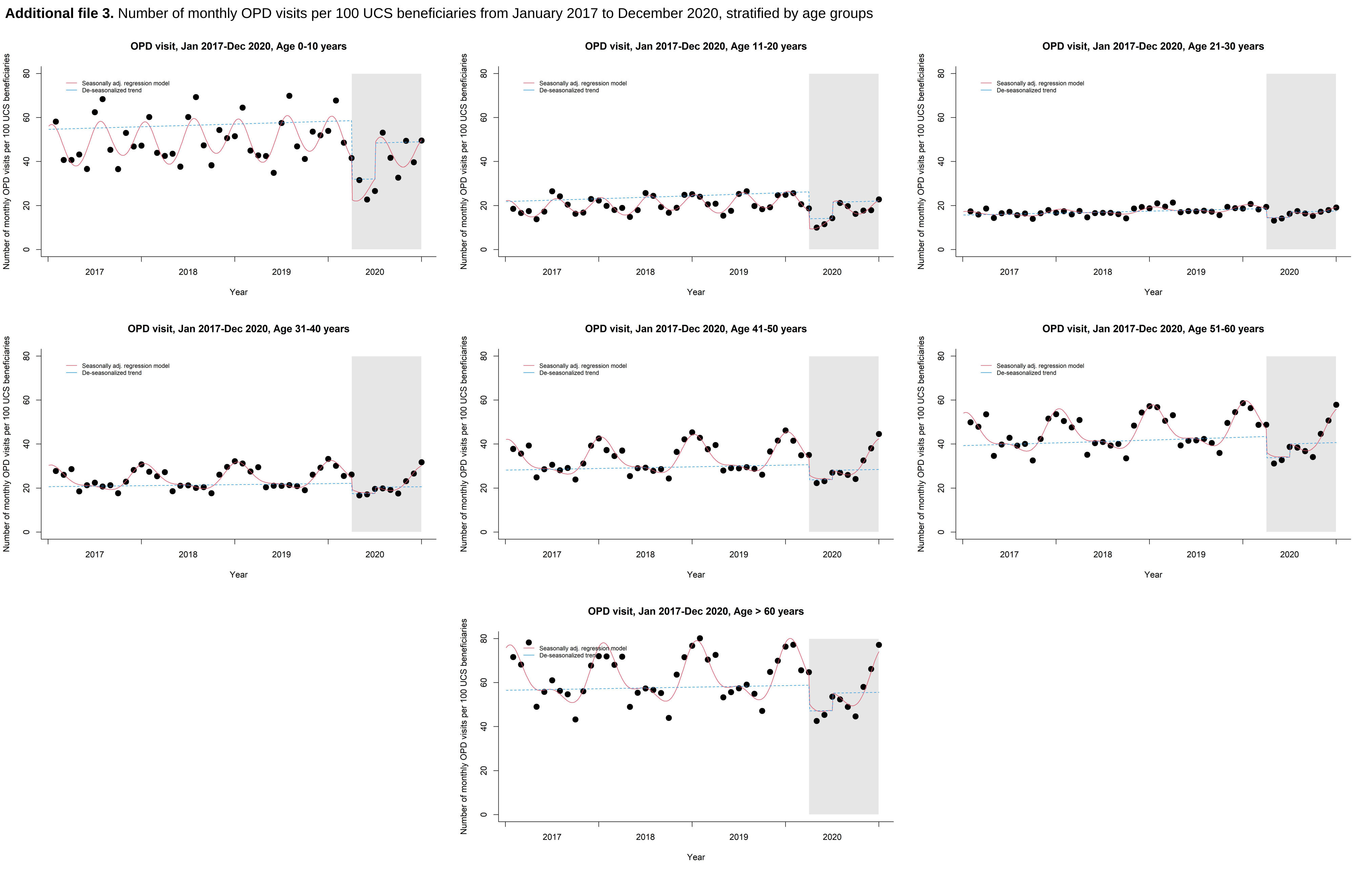

Additional file 3. Number of monthly OPD visits per 100 UCS beneficiaries from January 2017 to December 2020, stratified by age groups
