# Supplementary material for: Pattern of OPD utilisation during the COVID-19 pandemic under the Universal Coverage Scheme in Thailand: what can 850 million records tell us?
Source: BMC Health Serv Res. 2023 Feb 3;23:116. doi: 10.1186/s12913-023-09121-3 (PMC9897880; doi:10.1186/s12913-023-09121-3)
Supplement: Supplementary file 4 — Additional file 4: Number of monthly OPD visits per 100 UCS beneficiaries from January 2017 to December 2020, stratified by health regions. [file 12913_2023_9121_MOESM4_ESM.pptx]

## Slide 1
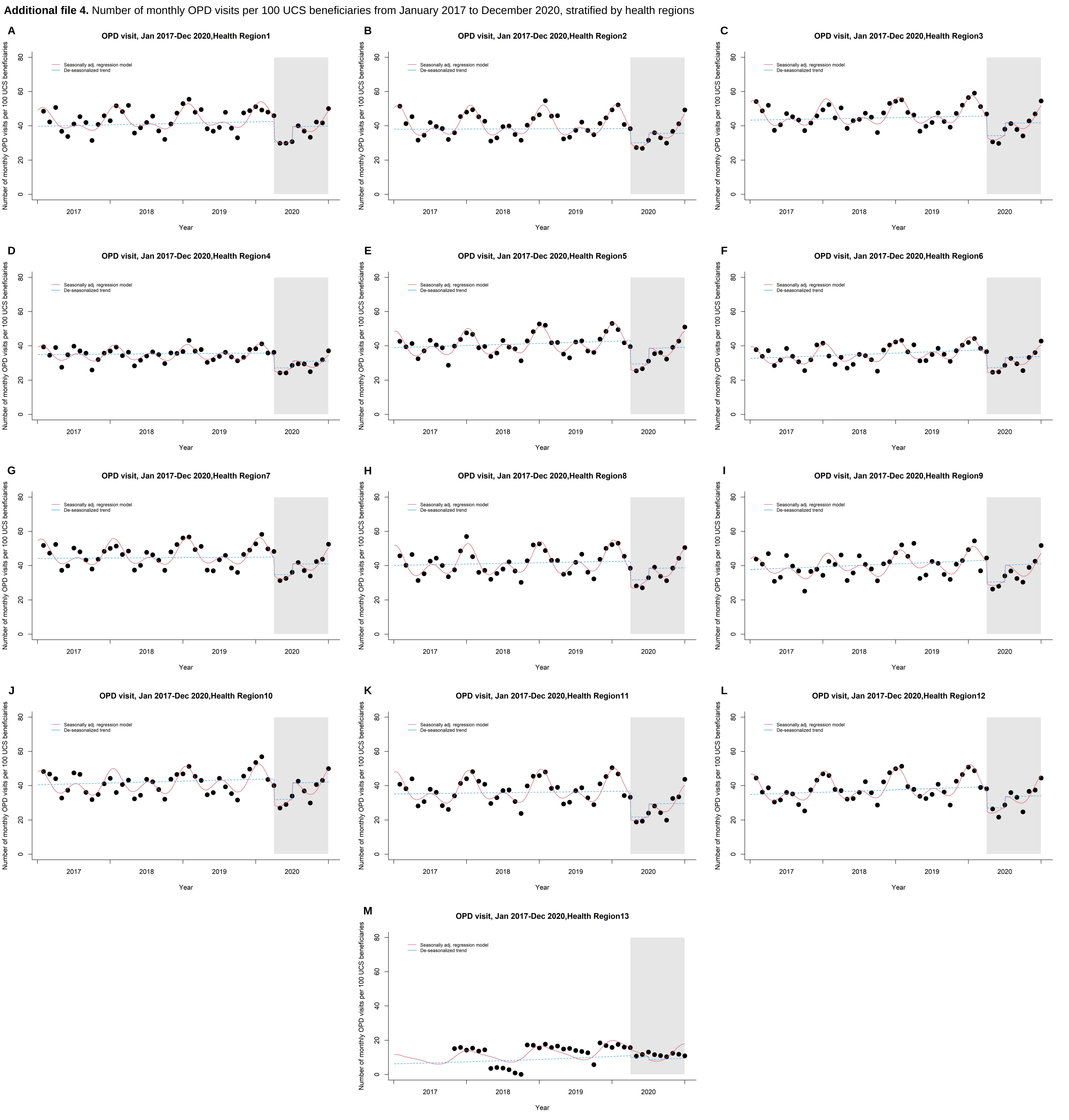

Additional file 4. Number of monthly OPD visits per 100 UCS beneficiaries from January 2017 to December 2020, stratified by health regions
C
A
B
D
E
F
G
I
H
J
K
L
M
